# Expert consensus on feasibility and application of automatic pain assessment in routine clinical use

**DOI:** 10.1186/s44158-025-00249-8

**Published:** 2025-06-02

**Authors:** Marco Cascella, Alfonso Maria Ponsiglione, Vittorio Santoriello, Maria Romano, Valentina Cerrone, Dalila Esposito, Mario Montedoro, Roberta Pellecchia, Gennaro Savoia, Giuliano Lo Bianco, Massimo Innamorato, Silvia Natoli, Jonathan Montomoli, Federico Semeraro, Elena Giovanna Bignami, Valentina Bellini, Matteo Luigi Giuseppe Leoni, Felice Occhigrossi, Alessandro Vittori, Maria Caterina Pace, Pasquale Buonanno, Mauro Forte, Elisabetta Chinè, Roberta Carpenedo, Alessandro De Cassai, Alfonso Papa, Maurizio Marchesini, Gaetano Terranova, Fabrizio Micheli, Laura Demartini, Franco Marinangeli, William Raffaeli, Flaminia Coluzzi, Andrea Tinnirello, Roberto Arcioni, Angelo Marra, Mohammed Naveed Shariff, Federica Monaco, Gabriele Finco, Alessia Bramanti, Ornella Piazza

**Affiliations:** 1 Department of Medicine, Surgery and Dentistry “Scuola Medica Salernitana”, Anesthesia and Pain Medicine, University of Salerno, Baronissi, Italy; 2 Department of Electrical Engineering and Information Technology, University of Naples Federico II, Naples, Italy; 3 Anesthesiology and Pain Department, Foundation G. Giglio Cefalù, Palermo, Italy; 4 Department of Neuroscience, AUSL Romagna, Pain Unit, Santa Maria Delle Croci Hospital, Ravenna, Italy; 5 Department of Clinical-Surgical, Diagnostic and Pediatric Sciences, University of Pavia, Pavia, Italy; 6 IRCCS Policlinco San Matteo, Pavia, Italy; 7 Division of Anesthesiology and Intensive Care, Infermi Hospital, AUSL Romagna , Rimini, Italy; 8 Department of Anesthesia, Intensive Care and Prehospital Emergency Maggiore Hospital Carlo Alberto Pizzardi, Bologna, Italy; 9 Department of Medicine and Surgery, Anesthesiology, Critical Care and Pain Medicine Division, University of Parma, Parma, Italy; 10 Department of Medical and Surgical Sciences and Translational Medicine, La Sapienza” University of Rome, Rome, Italy; 11 Pain Therapy Unit, San Giovanni-Addolorata Hospital, Rome, Italy; 12 Department of Anesthesia, Critical Care and Pain Medicine, ARCO, Ospedale Pediatrico Bambino Gesù IRCCS, Rome, Italy; 13 Department of Woman, Child and General and Specialized Surgery, University of Campania “Luigi Vanvitelli”, Naples, Italy; 14 Independent Scholar, Naples, Italy; 15 Unit of Pain Therapy, Polyclinic of Tor Vergata, Rome, Italy; 16 Department of Medicine (DIMED), University of Padua, Padua, Italy; 17 Institute of Anesthesia and Intensive Care, University Hospital of Padua, Padua, Italy; 18 Department of Pain Management, AO “Ospedale Dei Colli”, Monaldi Hospital, Naples, Italy; 19 Department of Anesthesia and Pain Medicine, Mater Olbia Hospital, Olbia, Italy; 20 Anaesthesia and Intensive Care Department, Asst Gaetano Pini, Milan, Italy; 21 Unit of Interventional and Surgical Pain Management, Guglielmo da Saliceto Hospital, Piacenza, Italy; 22 Pain Unit, IRCCS Maugeri, Pavia, Italy; 23 Department of Anesthesiology, Pain Treatment, Intensive and Palliative Care, University of L’Aquila, L’Aquila, Italy; 24 Institute for Research On Pain, ISAL Foundation, Rimini, Italy; 25 Department of Medical and Surgical Sciences and Biotechnologies, Unit of Anesthesia, Intensive Care and Pain Medicine, Sapienza University of Rome, Rome, Italy; 26 Anesthesiology and Pain Medicine Department, ASST Franciacorta, Ospedale Di Iseo, Iseo, Italy; 27 Sultan Qaboos Comprehensive Cancer Care and Research Centre (SQCCCR), Mascate, Oman; 28 Clinical Engineering, AOU San Giovanni Di Dio e Ruggi d’Aragona, Salerno, Italy; 29 Department of AI&DS, Rajalakshmi Institute of Technology, Chennai, Tamil Nadu India; 30 Department of Anesthesia, ASL Napoli 1, Naples, Italy; 31 Department of Medical Science and Public Health, University of Cagliari, Cagliari, Italy; 32 Department of Medicine, Surgery and Dentistry “Scuola Medica Salernitana”, University of Salerno, Baronissi, Italy

**Keywords:** Artificial intelligence, Automatic pain assessment, Pain, Pediatric pain, Opioid, Pain therapy, Pain medicine

## Abstract

**Background:**

Pain is often difficult to assess, particularly in non-communicative patients. While artificial intelligence (AI)-based objective Automatic Pain Assessment (APA) systems are a promising solution, their clinical implementation raises essential questions, primarily regarding clinician acceptance.

**Methods:**

We conducted a survey-to-consensus investigation on the feasibility and application of APA for clinical use. Firstly, the steering committee implemented the CHERRIES guidelines and designed a questionnaire for healthcare professionals. Given the survey results, 26 experts in pain medicine were asked to participate in a two-round consensus by rating 10 statements through a 7-point Likert scale. Consensus was defined as ≥ 75% agreement (“agree” or “completely agree”). For both phases, data was collected through online questionnaires and analyzed quantitatively.

**Results:**

For the survey, we collected responses from 628 healthcare professionals. The output highlighted excellent acceptance of the technology and a preference for multidimensional techniques. After two rounds, consensus was achieved on 8 out of 10 statements. Experts agreed on APA utility in supporting healthcare professionals and real-time pain monitoring. A strong consensus (96.2%) supported the need to inform patients about the use and limitations of AI systems. Adequate staff training is mandatory. Moreover, 92.3% agreed on the importance of implementing risk management, data quality control, and AI governance throughout the APA lifecycle. The experts stressed the need for internal and external validation processes and periodic updates, even for research purposes. Consensus was also reached about the importance of involving interdisciplinary stakeholders and addressing regulatory, ethical, and social implications. Multimodal inputs (e.g., physiological signals, facial expressions, speech, and clinical data) in APA systems are recommended. Additionally, APA systems should be capable of grading pain levels (e.g., via NRS), not just detecting the presence of pain. On the other hand, two statements did not reach consensus: the applicability of APA systems for acute and chronic pain conditions and their potential to improve therapeutic strategies.

**Conclusion:**

APA is viewed as a promising and potentially feasible technology for clinical pain assessment, particularly in vulnerable populations. Further research is needed to validate the dedicated tools, define applications in different clinical conditions (e.g., acute and chronic pain), and demonstrate their impact on routine clinical practice for pain management.

**Supplementary Information:**

The online version contains supplementary material available at 10.1186/s44158-025-00249-8.

## Background

Since pain is a complex and multidimensional experience influenced by a combination of several biological, psychological, and social factors [1], its accurate evaluation is often challenging, mostly in special populations, such as patients with cognitive impairments, non-verbal individuals [2–4], or those in intensive care [5], who may be unable to communicate their pain levels effectively.

Automatic Pain Assessment (APA) is a research field aimed at developing advanced technological solutions for pain evaluation [6]. Given the recent technological advancements, significant progress has been made toward the application of artificial intelligence (AI) methods for enhancing pain detection and monitoring [7]. These systems analyze a combination of physiological signals, such as heart rate and skin conductance [6], facial expressions [8, 9], language features [10], and other behavioral cues to provide a more objective and reliable assessment of pain [11, 12]. The goal is to integrate these technologies into routine clinical practice, supporting healthcare professionals in making timely and accurate pain management decisions [10–13].

Despite the potential benefits, the process of APA implementation raises important questions, especially regarding its acceptance among clinicians. The integration of AI-driven systems in medical decision-making could be accepted with skepticism, partly due to concerns about reliability, accuracy, and the role of human expertise in clinical judgment [14–16]. For instance, in a previous study focused on fibromyalgia, we observed that many healthcare professionals reported feeling unprepared to adopt AI-based technologies for disease management [17]. This gap highlights a broader issue of technological readiness and the need for structured education and training to facilitate the transition toward AI-supported healthcare [18, 19]. Furthermore, uncertainties persist regarding the practical applications of APA in different medical contexts. Healthcare professionals may question how these technologies can be effectively utilized across various domains of pain medicine, including acute and chronic pain management, oncologic and non-oncologic pain phenomena, and specific disease-related pain conditions. Additionally, APA systems could serve multiple functions, ranging from diagnosis and early detection of pain to treatment guidance, therapy personalization, and real-time patient monitoring. However, the specific roles these technologies should play and their clinical applicability remain topics of debate [11, 20, 21]. Moreover, the diversity of AI-driven tools being developed adds another layer of complexity. APA technologies can encompass a broad spectrum of solutions, from automated chatbots for patient-reported pain assessment to sophisticated diagnostic algorithms embedded in wearable devices or hospital monitoring systems [10]. Validation and standardization are crucial, as healthcare professionals must trust their accuracy, consistency, and ability to generalize across varied patient populations. Additionally, the ethical implications must be carefully considered, particularly regarding issues of patient autonomy, data privacy, bias in AI algorithms, and the potential dehumanization of medical care [22–25].

To address these concerns and explore the feasibility and acceptance of APA in clinical practice, we performed an expert consensus. The results could serve to guide the research focus in this uncharted area.

## Methods

The methodology for this study consisted of different key steps. A multidisciplinary team of experts, including clinicians, bioengineers, IT developers, stakeholders, ethicists (M.C., A.M.P., V.S., M.R., S.N, A.M., M.N.S., O.P.), was assembled to gather insights into the feasibility and application of APA in routine clinical practice.

### Clinical trial number

Not applicable.

#### Phase 1: cross-sectional web-based survey

The steering group assessed the evidence [26–28], met in person (December 2024; M.N.S. participated online), and developed a comprehensive questionnaire to assess current understanding, perspectives, and potential barriers related to the research aim. The Checklist for Reporting Results of Internet E-Surveys (CHERRIES) was implemented [29].

The research study’s version consists of 11 items. The questions from Q1 to Q4 refer to the APA benefits, and the questions Q5 and Q6 concern the preferred modality (unimodal or multimodal) of the APA system in daily clinical practice. These questions are 7-point Likert-Scale items ranging from “0 = completely disagree” to “6 = completely agree” to indicate the extent of the individual’s perception. Question Q7 is about the willingness to apply that device (“yes”, “no”, “perhaps”), and the further questions were for informational purposes (Appendix 1).

The questionnaire was distributed via Google Forms to diverse healthcare specialists, including clinicians, nurses, and other clinical staff. We shared the survey link via social media (LinkedIn), professional mailing lists, and direct invitations within relevant clinical and academic networks for 2 weeks. We targeted clinicians actively engaged in acute or chronic pain management, as well as professionals involved in pain-related research. Therefore, invitations were primarily distributed through professional networks, pain and anesthesiology associations, and academic mailing lists focused on these areas.

The survey was conducted anonymously via the SurveyMonkey platform, and the collected responses were systematically analyzed to identify common themes, challenges, and opportunities for implementing APA systems in clinical settings. Respondents were informed about the objectives of the study.

#### Phase 2: expert consensus

A modified Delphi method was implemented [30]. Based on data analysis, the steering committee panel designed the *Consensus Questionnaire* aimed at reaching an agreement on key aspects of APA, including its benefits, challenges, and implementation strategies.

For the consensus, the experts were selected based on their active involvement in scientific pain societies, scientific output, and their recognized clinical and academic expertise in the field of pain management. Participants were provided with educational materials on the topic, including textbooks, scientific articles, and links to informative websites.

The questionnaire consists of 10 questions (Appendix 1). Questions from Q1 to Q4 were about the APA approach in clinical applications, questions Q5 and Q6 referred to the level of knowledge of the user, questions from Q7 to Q9 concerned technical and legal issues, and question Q10 asked whether the APA approach must necessarily provide a pain severity scale. Each question was a 7-point Likert scale ranging from “0 = completely disagree” to “6 = completely agree” followed by a white space where notes could be entered. The consensus was defined as at least 75% agreement (“agree” or “completely agree”) among those who responded [31]. Those who chose not to answer to a particular statement, were excluded from the consensus for that statement. This survey was designed by Google Form and then shared online with the experts via the SurveyMonkey platform. Each participant previously read a presentation letter and then filled out the informed consent. The survey was conducted anonymously.

**Fig. 1:**
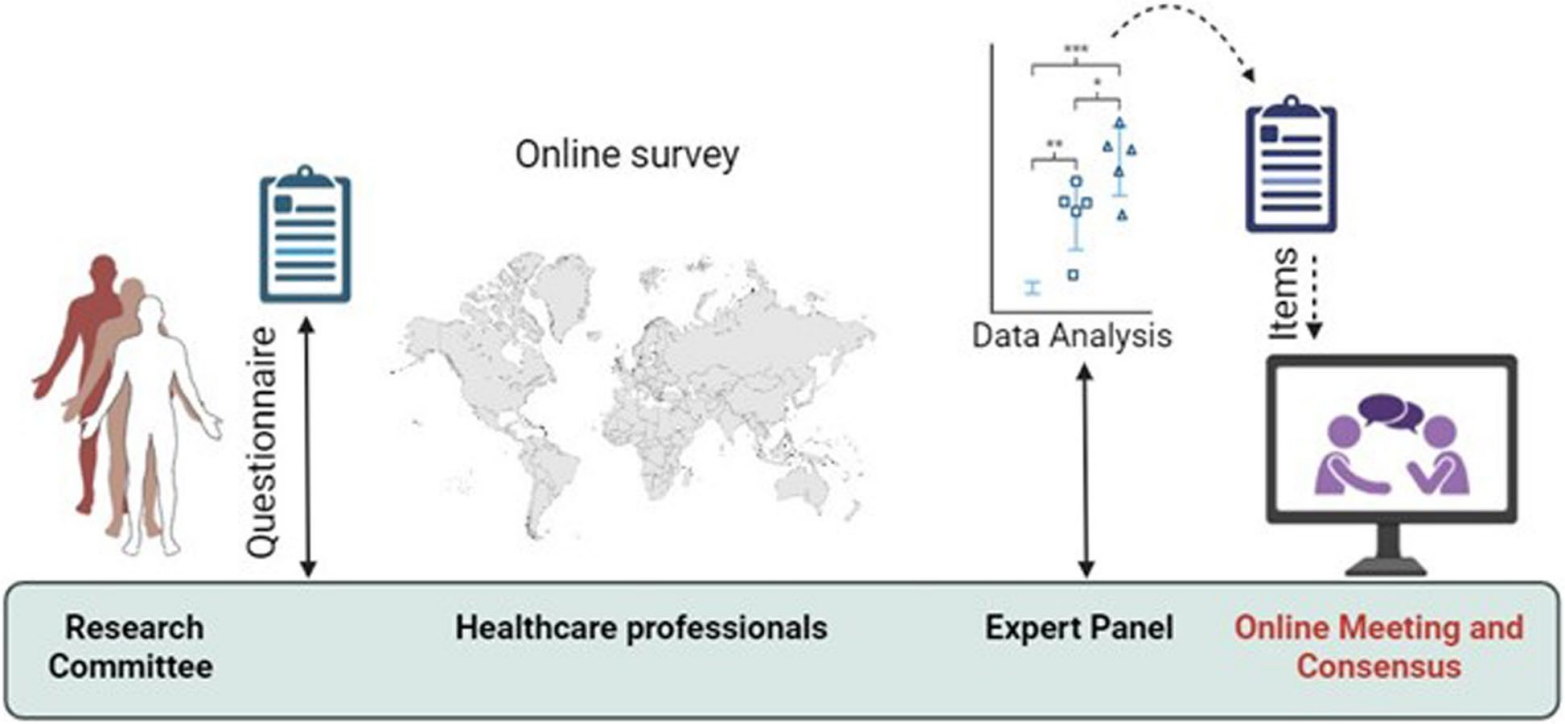
Study framework

Finally, an online meeting was held to discuss the findings, review the data, and finalize the consensus. During this meeting, experts were able to clarify any ambiguities and ensure that all key issues were addressed before concluding the study (Fig. 1).

Ethical review and approval were waived for this survey, which did not explore individual patient therapies or pathologies, nor did it collect sensitive personal or clinical data. According to Italian legislation for non-interventional studies (Ministerial Circular N. 6, 2 September 2002), ethics committee approval is only required if the study addresses issues related to prescribed medicinal products, where patient inclusion in therapeutic strategies is determined by normal clinical practice rather than the trial protocol.

#### Statistical analyses and data processing

The dataset was collected in an Excel spreadsheet. Statistical analysis was carried out by implementing the IBM SPSS Statistics software (version 29).

An exploratory data visualization and analysis was conducted by looking at bar plots and boxplots showing frequency of responses for each item in the questionnaire to visually examine the distribution of the results across the 7-point Likert-scale. Then, to compare responses regarding APA benefits (questions Q1-Q2-Q3-Q4 of the first questionnaire) across multiple paired groups, the non-parametric Friedman test was conducted, followed by a post hoc Wilcoxon test with Bonferroni Correction. Additionally, the Mann–Whitney test was employed to assess differences in responses regarding APA benefits between gender groups (female vs. male) and age groups (under 40 vs. over 40). Furthermore, the Kruskal–Wallis’s test was applied to evaluate differences in benefits across various job categories (physicians vs. nurses vs. other), with significant results being further examined using post hoc Signed-Ranked Wilcoxon test to identify specific group differences. The preference for either unimodal or multimodal APA approaches (questions Q5-Q6 of the first questionnaire) was then analysed through a non-parametric Wilcoxon test. For all the adopted statistical tests a significance level alpha of 0.05 was chosen (95% confidence interval).

The Cronbach’s alpha was calculated for the entire survey to determine its overall reliability. Additionally, it was calculated separately for each of the two dimensions (APA benefits and APA modalities) to assess the reliability of the items within each dimension. We considered a reliability level as excellent for a Cronbach’s Alpha Range of 0.90 and above (i.e., very high internal consistency level); good (Cronbach’s 0.80–0.89, strong internal consistency level); acceptable (Cronbach’s 0.70–0.79, acceptable internal consistency level); questionable (Cronbach’s 0.60–0.69, questionable internal consistency); and poor (Cronbach’s < 0.60, poor internal consistency).

## Results

### Cross-sectional investigation

Regarding the first explorative questionnaire, responses from 628 participants were collected (327/52% women, 301/48% men). We analyzed responses from clinicians (*n* = 427, 68%), nurses (*n* = 113, 18%), and other healthcare personnel such as clinical psychologists, physical therapists, and occupational therapists (*n* = 88, 14%). The average age was 43.8 years (SD = 11.9). Participants were further divided into two main groups according to age: 260 people (41%) were under 40 (≤ 40 years old) and 368 (59%) were over 40 (> 40 years old). Most respondents were from Europe (*n* = 614, 97.8%) (Table 1).

**Table 1:** Socio-demographic descriptive statistics of the respondents (*n* = 628)

Variable	*n*	%
Gender		
**Women**	327	52%
**Men**	301	48%
Professional		
**Clinicians**	427	68%
**Nurses**	113	18%
**Other**	88	14%
Age		
**Under 40**	260	41%
**Over 40**	368	59%
Nationality		
**European**	614	97.8%
**Asian**	7	1%
**American**	4	1%
**African**	2	< 1%
**Australian**	1	< 1%

Clinical psychologists, physical therapists, occupational therapists

**Fig. 2:**
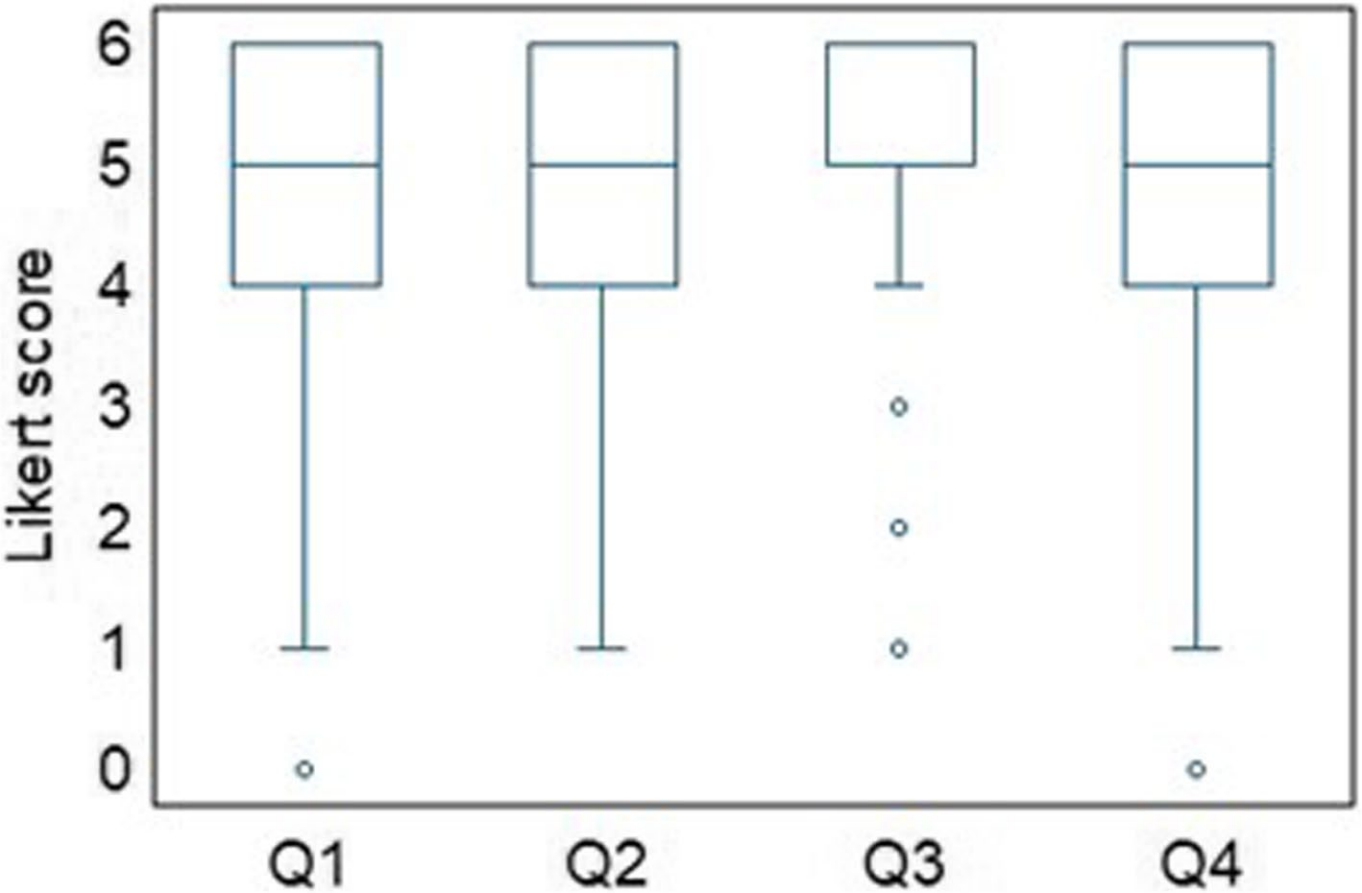
Potential advantages of Automatic Pain Assessment in different settings. Legend: Boxplots from Q1 to Q4 (Q1 “intensive care settings”, Q2 “complications detection”, Q3 “analgesic dosage”, Q4 “inpatients management at night”)

Results indicated a positive perception of APA benefits in diverse settings and context of application, with medians (and interquartile range, IQR) respectively of 5 (IQR = 2) for Q1, 5 (IQR = 2) for Q2, 6 (IQR = 1) for Q3, and 5 (IQR = 2) for Q4. In particular, question Q3 “Automatic pain assessment provides a benefit in terms of preventing over- or undersupply of analgesics for patients with limited communicative abilities” reached the highest median (equal to 6, i.e., “completely agree”), even though a few outliers were found (Fig. 2).

Overall, the results suggest a generally positive perception of benefits, with median scores clustering toward the higher end of the scale, though some variability is observed due to a few outliers. The Friedman test confirmed a significant difference across the ratings, given by the respondents across the first four questions regarding the APA benefits (*p*-value 1.108∙10^−13^).

The post-hoc pairwise comparison carried out through Dunn’s tests with Bonferroni correction highlighted a statistically significant difference, although with small effect size, between Q1 and Q3 (*p*-value = 0.002, standardized test statistics = − 3.574; *n* = 628; effect size = 0.143), Q2 and Q3 (*p*-value = 1.744∙10^−5^, standardized test statistics = − 4.677; *n* = 628; effect size = 0.187) and Q3 and Q4 (*p*-value = 2.045∙10^−5^, standardized test statistics = 4.645; *n* = 628; effect size = 0.185), thus confirming Q3 (benefits of using APA for better analgesic management and dosage) as the most relevant benefit of APA systems according to the respondents’ opinion.

**Fig. 3:**
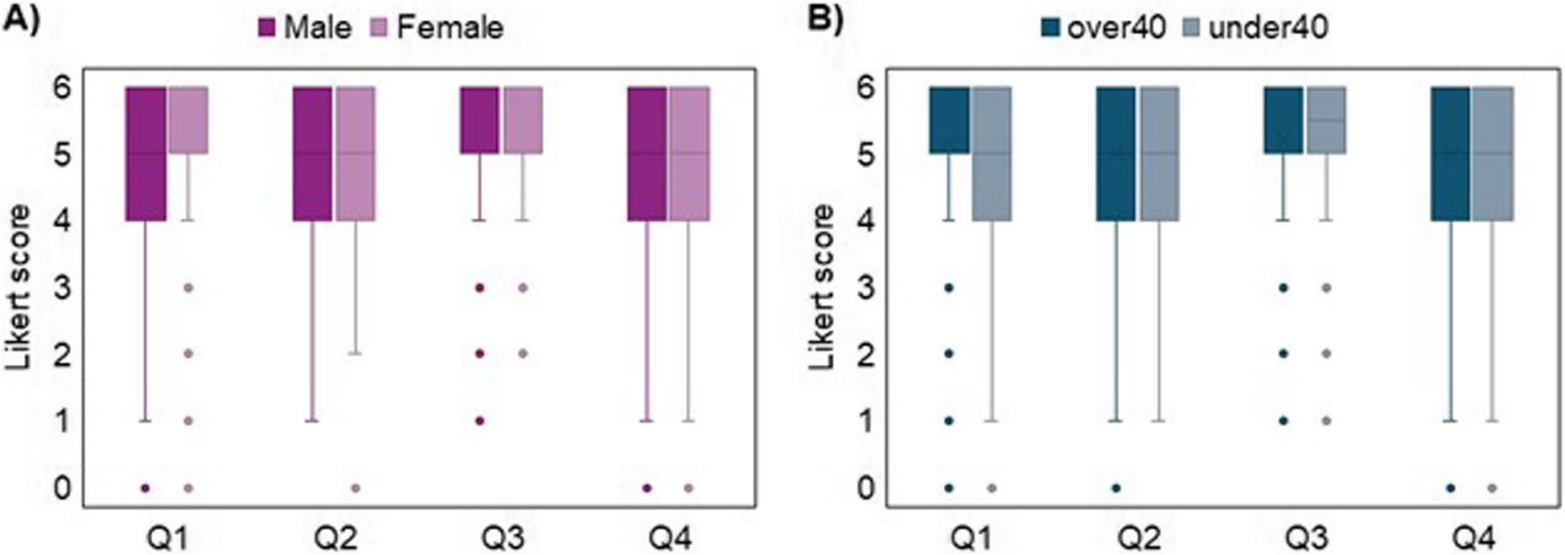
Different settings for Automatic Pain Assessment implementation. Legend: Boxplots Likert scale scores from Q1 to Q4 of the questionnaire grouped by gender (**A**) and age (**B**) (Q1 “intensive care settings”, Q2 “complications detection”, Q3 “analgesic dosage”, Q4 “inpatients management at night”)

Subgroups analysis by gender and age did not show particular tendency; indeed, the non-parametric Mann Whitney test for each of the Q1-Q2-Q3-Q4 questions failed to demonstrate a significant influence of gender and age (*p*-values for gender and age groups were respectively: 0.540 and 0.03 for Q1, 0.430 and 0.089 for Q2, 0.969 and 0.510 for Q3, 0.540 and 0.247 for Q4), exception made for Q1 when grouped by age, which demonstrated a slightly higher score, with a small effect size (standardized test statistics = − 2.987; *n* = 628; effect size = 0.119), in the over40 group. Boxplots of the Likert scale scores given by the respondents are reported in Fig. 3.

The Reliability Analysis for APA benefits pointed out that Cronbach’s Alpha was 0.857, which means that the items consistently measured the respondents’ degree of agreement on APA benefits.

**Fig. 4:**
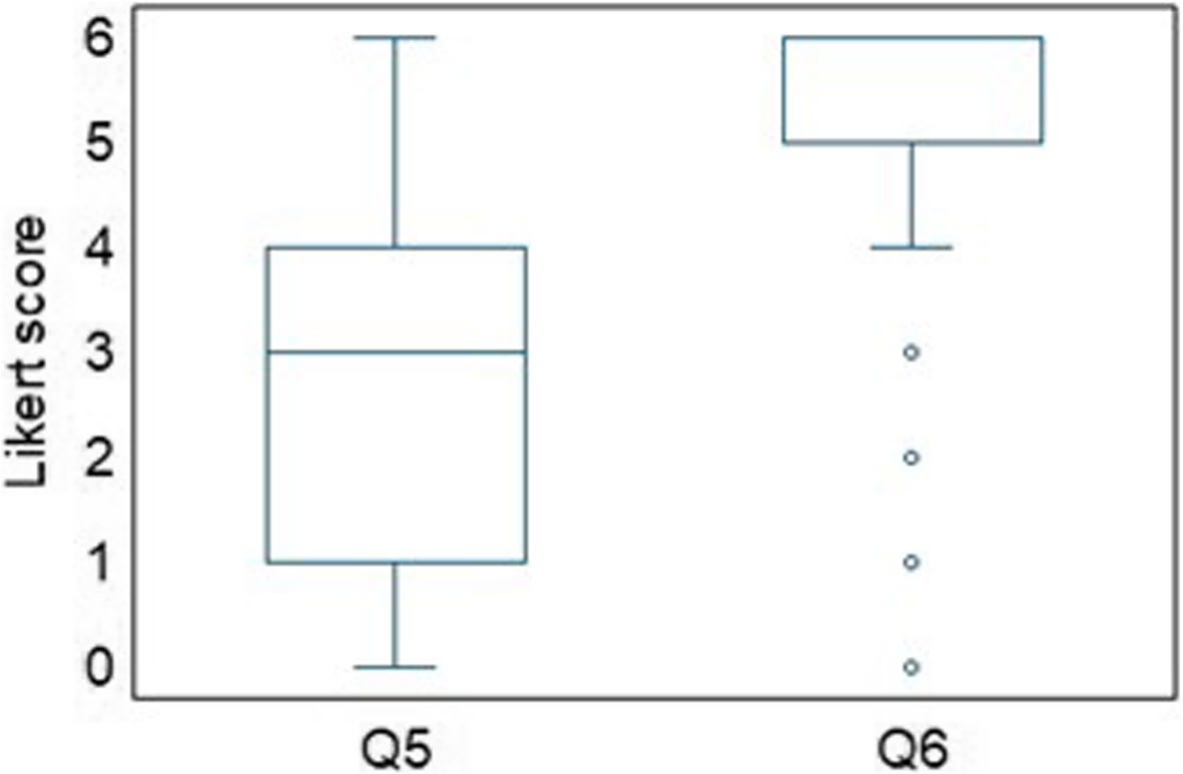
Different approaches to Automatic Pain Assessment. Legend: Boxplots Likert scale scores to questions Q5 and Q6 of the questionnaire (Q5 “unimodal approaches”, Q6 “multimodal approaches”)

Questions Q5 and Q6 investigated the modality (unimodal or multimodal) of APA utilization that the medical personnel predominantly favoured. The preferred expected modality was that “Automatic pain assessment should be realized using a multimodal approach (e.g., using simultaneously physiological, (para)linguistic, video-based parameters)”. The median in Q6 was 5 and the limited IQR indicates a lower variability in responses. Most respondents expressed strong agreement that the multimodal approach was the most effective option for implementing the APA system (Fig. 4).

**Fig. 5:**
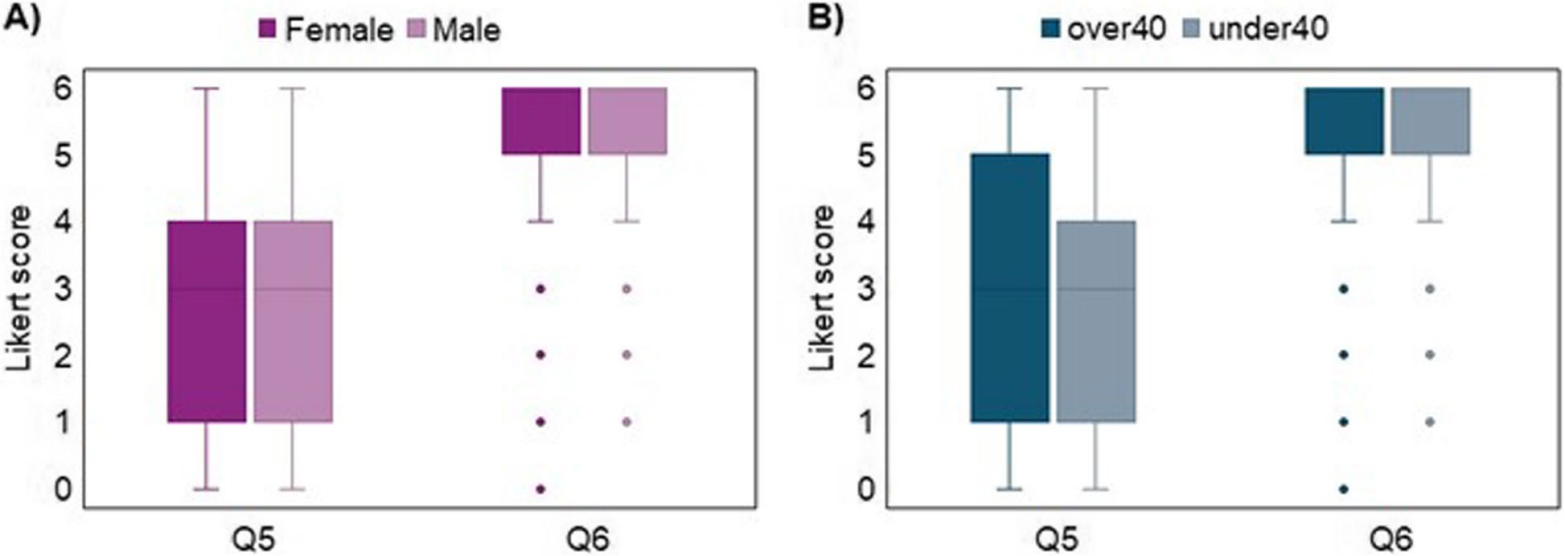
Different modalities for Automatic Pain Assessment implementation. Legend: Boxplots Likert scale scores from Q1 to Q4 of the questionnaire grouped by gender (**A**) and age (**B**) (Q5 “unimodal approaches”, Q6 “multimodal approaches”)

**Fig. 6:**
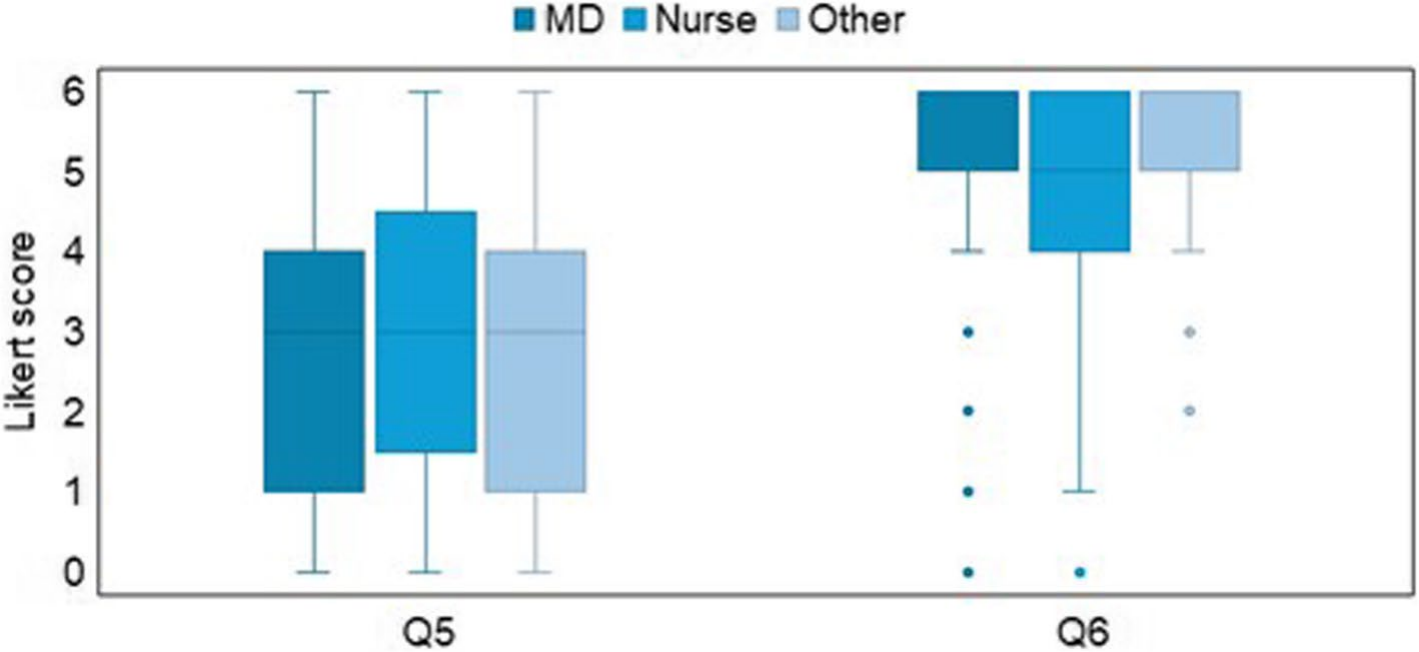
Different modalities for Automatic Pain Assessment implementation. Legend: Boxplots Likert scale scores from Q5 to Q6 of the questionnaire grouped by healthcare profession (MD, Nurse, Other) (Q5 “*unimodal approaches*”, Q6 “*multimodal approaches*”)

Results indicated a positive perception towards multimodal approaches to implement APA (question n. 6: median = 5, IQR = 1) compared to unimodal approaches (question n. 5: median = 3, IQR = 3). The non-parametric Wilcoxon signed-rank test confirmed a significant difference between the two approaches (*p*-value = 0.000) with an almost large effect size of 0.721 (standardized test statistics = 18.067; *n* = 628). Regarding gender and age (Fig. 5) and healthcare profession (Fig. 6), there was no statistical significance among the two modalities of APA approaches; indeed, the non-parametric Mann–Whitney test for each of the Q5–Q6 questions failed to demonstrate a significant influence of gender and age (*p*-values for gender and age groups were respectively: 0.075 and 0.202 for Q5, 0.972 and 0.995 for Q6) as well as of healthcare profession (*p*-values for gender and age groups were respectively: 0.562 for Q5, 0.156 for Q6).

**Fig. 7:**
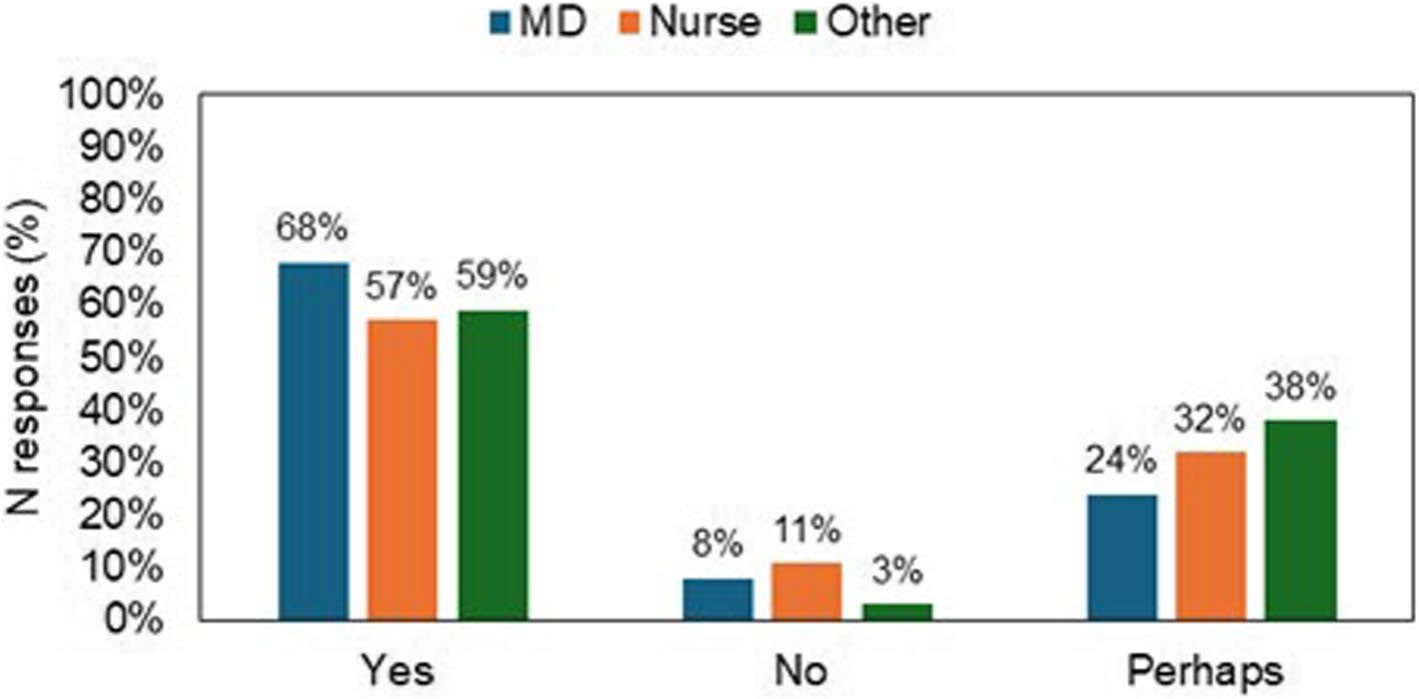
Propensity to use Automatic Pain Assessment tools in clinical practice. Legend: Bar chart of Likert scale scores to question Q7 grouped by healthcare profession

Finally, question Q7 investigated how prone healthcare professionals are, based on their profession, to use APA systems in clinical practice (Fig. 6). It resulted that a total of 65% (of which, 68% of MDs, 57% of nurses, 59% of other professions) of the respondents would use it, 8% (of which, 8% of MDs, 11% of nurses, 3% of other professionals) would not use it, and 27% (of which, 24% of MDs, 32% of nurses, 38% of other professions) answered “perhaps” (Fig. 7).

### Expert consensus

From the cross-sectional investigation, literature assessment, and input from the participants and experts, different key issues were recognized:

- Interindividual variability in pain expression complicates the consistency of automated assessments.
- AI-based technologies lack robust clinical validation and universally accepted standards, which undermines their reliability in real-world settings.
- It is essential to understand in which contexts of acute and chronic pain, and in which clinical settings, this technology can be effectively utilized.
- Another concern involves the use of APA to inform therapeutic decision-making.
- APA implementation requires substantial technical and financial barriers due to the application of hardware and advanced software infrastructure.
- The clinical acceptance of these tools may be hindered by skepticism among healthcare professionals and the need for targeted training, especially in complex or ambiguous situations.
- Specific training is required to integrate these tools into the clinical workflow.
- The applicability of these systems is often limited to controlled environments, with uncertain performance in complex or dynamic clinical contexts, especially among patients with neurological or cognitive impairments.
- Ethical and privacy concerns also arise, particularly regarding the collection and storage of sensitive biometric data.

The expert consensus was launched. After the first round, 8 recommendations reached the required level of agreement (75%). Two items “Automatic pain assessment can be applied for acute (e.g., procedural pain in individuals with limited communication skills) and chronic pain problems” (65.4%) and “Automatic pain assessment provides an advantage in terms of therapeutic improvement (intended for drug therapies and methods with different invasiveness)” (73.1%) were revised based on participant feedback, and a second round was conducted. The results are presented in Table 2.

**Table 2:** Expert consensus on feasibility and application of automatic pain assessment in routine clinical use

	***N* responses (5 and 6 points on the Likert scale)**	***N* responses (%)**
Q1. Artificial intelligence for automatic pain assessment can be applied effectively in acute pain management (e.g., in procedural pain in patients with limited communication skills) and in chronic pain monitoring and treating chronic pain	19/26	73.1%^a^
*Q2.* Automatic pain assessment offers an advantage for healthcare professionals and patients, for real-time pain monitoring and after processing the collected multimodal elements (e.g., after an analysis of a patient video)	20/26	76.9%
Q3. The use of artificial intelligence for the automatic assessment of pain can contribute to an improvement in the therapeutic approach, whether pharmacological or through interventions with different invasiveness	19/26	73.1%^a^
*Q4.* The patient must be aware of the use of AI techniques (for diagnostic and/or therapeutic purposes) and their application limitations (e.g., accuracy not 100%) and must be provided with documentation/clinical explanations	25/26	96.2%
*Q5.* Those who use automatic pain assessment (like hardware, application or other modalities) for research must implement a risk management process throughout the AI life cycle, define mechanisms for input and output quality control (including data security), adopt a system for periodic audits and updates, record usage through a logging system and establish effective mechanisms for AI governance (including human-AI interactions)	24/26	92.3%
*Q6.* The clinical use of automatic pain assessment (like hardware, application, or other modalities) must necessarily include training	25/26	96.2%
*Q7.* Automatic pain assessment should be realized using tools developed on multimodal elements, combining physiological parameters (e.g., EMG, ECG, EDA), speech and facial expression analysis (e.g., from video), and clinical data	22/26	84.6%
*Q8.* The development of AI systems for automatic pain assessment must involve interdisciplinary stakeholders throughout the entire AI lifecycle, implement data privacy and security measures, adopt strategies to mitigate identified risks, define an appropriate evaluation plan based on datasets, metrics, and benchmark methods, ensure compliance with applicable regulatory requirements, address application-specific ethical issues, and consider the societal implications of AI use	23/26	88.5%
*Q9.* The development of AI systems for automatic pain assessment must include thorough internal (explainable AI) and external validation on datasets or in the real world, with periodic updates. This also applies to research purposes	24/26	92.3%
*Q10.* An automatic pain recognition system, in addition to distinguishing between the presence and absence of pain (pain versus non-pain category), must necessarily be able to define pain levels (e.g., corresponding to the NRS scale)	20/26	76.9%

*Abbreviations*: *APA* Automatic pain assessment, *EMG* Electromyography, *EDA* Electrodermal activity

^a^After 2 rounds

## Discussion

A growing number of studies investigated APA by using AI models developed on different physiological biosignals and behavioral strategies [6–13, 26–28]. However, the expectations, requirements, and acceptance of these technologies for practical use in pain medicine must be carefully assessed. To address this need, the consensus process described in this study was specifically designed to guide the development of more calibrated research projects in this emerging field.

In a previous qualitative study, Walter et al. [32] developed a 15-item questionnaire on APA. After watching a video about APA technology, participants (*n* = 102) expressed their opinion about the potential benefits in medical practice of these methods. In our study, we employed a more comprehensive methodology that included the involvement of different professional healthcare categories, followed by consultation with pain management experts. This survey-to-consensus strategy can be particularly suitable for addressing a poorly defined or evolving topic to help establish clear recommendations [33].

From the preliminary comprehensive analysis (survey), we found a positive perception of benefits (Q1–Q4) without differences across gender and age. Furthermore, participants expressed a significant preference for the multimodal APA approach (Q5 and Q6). Although gender and age were not significant predictors, we found a slight preference among female and younger (< 40-year-old) participants.

Concerning the expert consensus, the high level of agreement reached on most items reflects a strong belief that APA systems are technically feasible. Nonetheless, the experts emphasized that these approaches must adhere to ethical standards, especially when involving vulnerable or non-communicative patient groups.

Notably, although the panel recognized the potential of APA in enhancing pain recognition across both acute and chronic settings, after two rounds, the item did not reach a consensus. In our opinion, this lack highlights the complexity of generalizing such tools. Consequently, there is a need for the careful tailoring of APA systems to distinguish clinical contexts rather than proposing universal applicability. Research must address crucial issues such as in which acute or chronic pain scenarios APA should be applied. In their comments, the experts underscored the distinction between nociception and pain, a difference that becomes particularly relevant in chronic pain, where biopsychosocial factors significantly shape the pain experience. These challenges highlight why applying AI to the study of pain remains such a complex and demanding task [34]. For example, machine learning and deep learning models have shown promising results in identifying pain markers, particularly in acute pain scenarios where physiological signals can be highly reliable [35, 36]. Additionally, different investigations have been conducted by implementing datasets collecting information on acute pain [37, 38]. However, the application of AI in chronic pain remains limited. Unlike acute pain, chronic pain lacks consistent predictive models and involves dimensions—such as emotional and cognitive components—that are difficult to quantify [39, 40]. Primary chronic pain (e.g., fibromyalgia) and complex cancer pain phenomena, such as breakthrough pain [9], further challenge traditional models and demand dedicated AI strategies. Therefore, the multifaceted nature of pain, combined with algorithmic limitations in complex clinical scenarios and the lack of longitudinal data, highlights a significant gap in this field of research. At the same time, these challenges offer a fascinating opportunity for future investigation and innovation.

Similarly, the item addressing the potential therapeutic benefits of APA (e.g., enabling more accurate or timely pharmacological interventions) narrowly missed the threshold for consensus in its original formulation. The revised version focused on the APA’s role in supporting, rather than directing, therapeutic choices. Nevertheless, a consensus was not reached (73%). This highlights a key issue: Clinicians still view APA as an adjunct rather than a standalone decision-making tool. The distinction is crucial in maintaining trust in human oversight while integrating AI technologies into sensitive domains like pain management. A clear involvement of the human-in-the-loop during model development, along with verification of performance and outcomes through explainable AI processes, will likely foster greater trust among end users [41]. These aspects also received full agreement, reaching over 90% consensus (Q5 and Q9) and align with emerging international frameworks for trustworthy AI, such as the European Union AI Act and the U.S. FDA’s digital health guidelines, which emphasize transparency, accountability, and risk management in clinical AI applications [42, 43].

To enhance AI applications in pain assessment, a multimodal approach is essential. Integrating traditional clinical evaluations, patient-reported outcomes, and different APA data can provide a more comprehensive and accurate picture. Key questions persist: which data combinations are most effective for which patient groups, and in what clinical context?

Furthermore, the development of robust AI systems for pain requires interdisciplinary collaboration between clinicians, data scientists, and bioengineers. This collaboration is crucial not only for technical development but also for addressing concerns related to data quality, explainability of AI decisions, and ethical implications—especially when dealing with vulnerable populations such as pediatric or non-verbal patients [2]. The consensus, indeed, pointed toward significant ethical, legal, and user-related considerations. Moreover, the findings point to a clear role for scientific societies and interdisciplinary collaborations. Pain specialists, ethicists, data scientists, and AI-literate clinicians must work together from the early stages of APA development—including dataset creation, preprocessing, and modeling—to ensure that future tools are not only technologically sound but also clinically meaningful and ethically acceptable. Equally emphasized was the importance of usability: APA systems must be designed with clinicians and patients in mind, ensuring intuitive interfaces and seamless integration into existing workflows.

Despite encouraging agreement on many points, the Delphi results also reveal that the more complex the issue, the harder it is to achieve full consensus. This is particularly evident in statements involving APA’s impact on treatment choices or its capacity to replace subjective human assessment. These areas merit further study and suggest that external validation through real-world trials is essential before widespread clinical adoption.

### Limitations

Although the panel conducted a preliminary literature review, most available studies primarily focus on the methodological aspects of APA, with limited high-quality clinical evidence. Additionally, the literature showed significant gaps concerning key issues related to ethics and real-world validation. Therefore, the adopted methodology (survey-to-consensus) was chosen due to the lack of robust evidence on the topic and to ensure that the consensus was grounded in real-world needs rather than solely on theoretical assumptions.

Concerning the survey, due to the open distribution strategy employed—primarily via professional mailing lists and social media platforms—it was not possible to determine the exact number of individuals who received the survey invitation. As a result, calculating an accurate response rate is challenging. Nevertheless, while this limits the ability to assess selection bias, the overall sample size of 628 respondents remains substantial and supports the robustness of the descriptive and thematic findings. Another limitation of the survey is the geographic distribution of respondents, with 97.8% working within European healthcare settings. This regional predominance may influence the generalizability of our findings, as perceptions of the feasibility, utility, and ethical acceptability of AI-based APA systems could differ significantly in non-European or lower-resource contexts. Variations in digital infrastructure, healthcare priorities, and cultural attitudes toward technology may shape stakeholder perspectives in ways not captured by our current sample. Future studies should aim to include a more geographically diverse cohort to enhance external validity.

## Conclusions

The expert consensus gathered in this study highlights the perceived value and feasibility of AI-based APA in clinical practice. These tools are especially relevant for populations where conventional pain reporting is challenging, such as patients in intensive care or with cognitive impairments. Nevertheless, for widespread adoption, the APA must meet high standards of accuracy, explainability, and data privacy. Integration into existing clinical systems and workflows must be seamless and supported by adequate training and stakeholder engagement.

Future research should focus on validating the APA in real-world clinical environments and exploring its impact on patient outcomes, workflow efficiency, and ethical acceptability. The collaborative involvement of healthcare professionals, AI experts, and policymakers will be essential to ensure responsible and effective implementation. Research is also mandatory for better defining applications in different clinical settings (acute vs chronic pain) and for regulating the role of technology for guiding pain management.

## Supplementary Information

Supplementary Material 1: Appendix 1. Consensus questionnaire.

## Data Availability

Availability of data and materials: The datasets used and/or analysed during the current study are available from the corresponding author on reasonable request.

## Abbreviations

AI: Artificial Intelligence
APA: Automatic Pain Assessment
CHERRIES: Checklist for Reporting Results of Internet E-Surveys
ECG: Electrocardiogram
EDA: Electrodermal activity
EMG: Electromyography
NRS: Numeric Rating Scale
VIF: Variance Inflation Factor
