# Supplementary material for: Expert consensus on feasibility and application of automatic pain assessment in routine clinical use
Source: J Anesth Analg Crit Care. 2025 Jun 2;5:29. doi: 10.1186/s44158-025-00249-8 (PMC12131339; doi:10.1186/s44158-025-00249-8)
Supplement: Supplementary file 1 — Supplementary Material 1: Appendix 1. Consensus questionnaire. [file 44158_2025_249_MOESM1_ESM.docx]

**PHASE 1. Healthcare personnel’s perception of APA system**

Q1. Automatic pain assessment provides a benefit in acute medical settings (e.g. intensive care) for healthcare staff and patients.

Q2. Automatic pain assessment provides a benefit as a diagnostic tool in the detection of complications for healthcare staff and patients.

Q3. Automatic pain assessment provides a benefit in terms of preventing over- or undersupply of analgesics for patients with limited communicative abilities.

Q4. Automatic pain assessment provides benefits for healthcare staff and patients during inpatient stays at night.

Q5. Automatic pain assessment should be realized using an unimodal approach (e.g. using individually physiological, (para)linguistic, video-based parameters).

Q6. Automatic pain assessment should be realized using a multimodal approach (e.g. using simultaneously physiological, (para)linguistic, video-based parameters).

Q7. Would you personally use such a device?

Q8. What is your gender?

Q9. How old are you?

Q10. What is your job? Specify the clinical field you belong to.

Q11. Where are you from?

**PHASE 2. Expert consensus**

Q1. Automatic pain recognition can be applied for acute (e.g., in procedural pain in individuals with limited communication skills) and chronic pain problems. NB: in case of disagreement, please express the rationale in the Notes.

| APPENDIX 1 |
| --- |

*Second Round Q1. Artificial intelligence for automatic pain recognition can be applied effectively in acute pain management (e.g., in procedural pain in patients with limited communication skills) and in chronic pain monitoring and treating chronic pain.*

Q2. Automatic pain recognition offers an advantage for healthcare professionals and patients, both for real-time pain monitoring and after processing of the collected multimodal elements (e.g., after an analysis of a patient video).

Q3. Automatic pain recognition provides an advantage in terms of therapeutic improvement (intended for drug therapies and methods with different invasiveness).

*Second Round Q3. The use of artificial intelligence for the automatic recognition of pain can contribute to an improvement in the therapeutic approach, whether pharmacological or through interventions with different invasiveness.*

Q4. The patient must be aware of the use of AI techniques (for diagnostic and/or therapeutic purposes) and of their application limitations (e.g., accuracy not 100%) and must be provided with documentation/clinical explanations.

Q5. Those who use APA systems (as hardware, application or other modalities) for research must implement a risk management process throughout the AI life cycle, define mechanisms for input and output quality control (including data security), adopt a system for periodic audits and updates, record usage through a logging system and establish effective mechanisms for AI governance (including human-AI interactions).

Q6. The clinical use of APA systems (as hardware, application or other modalities) must necessarily include training.

Q7. Automatic pain recognition should be realized using tools developed on multimodal elements, combining physiological parameters (e.g., EMG, ECG, EDA), speech and facial expression analysis (e.g., from video), and clinical data.

Q8. The development of AI systems for pain must involve interdisciplinary stakeholders throughout the entire AI lifecycle, implement data privacy and security measures, adopt strategies to mitigate identified risks, define an appropriate evaluation plan based on datasets, metrics, and benchmark methods, ensure compliance with applicable regulatory requirements, address application-specific ethical issues, and consider the societal implications of AI use.

Q9. The development of AI systems for pain must include thorough internal (explainable AI) and external validation on datasets or in the real world, with periodic updates. This also applies for research purposes.

Q10. An automatic pain recognition system, in addition to distinguishing between the presence and absence of pain (pain versus non-pain category), must necessarily be able to define pain levels (e.g. corresponding to the NRS scale).
